# Sequencing for an interdisciplinary molecular tumor board in patients with advanced breast cancer: experiences from a case series

**DOI:** 10.18632/oncotarget.27704

**Published:** 2020-09-01

**Authors:** Christina Walter, Andreas Hartkopf, Andre Koch, Marion Klaumünzer, Martin Schulze, Eva-Maria Grischke, Florin-Andrei Taran, Sara Brucker, Florian Battke, Saskia Biskup

**Affiliations:** ^1^Department of Women’s Health, University of Tuebingen, Tuebingen, Germany; ^2^Praxis fuer Humangenetik and CeGaT GmbH, Tuebingen, Germany

**Keywords:** breast cancer, genetics, next-generation sequencing, actionable mutations

## Abstract

Purpose: High throughput panel sequencing to tailor therapy in precision oncology promises to improve outcome in patients with metastatic breast cancer. However, data that clearly show any benefit from such an approach is still pending.

Materials and Methods: We performed a retrospective analysis of advanced breast cancer patients that underwent panel sequencing for suggestion of target related drugs. We aimed to (i) determine the frequency of actionable mutations per patient and to (ii) assess the clinical impact of results on treatment options.

Results: A total of 52 patients underwent panel sequencing of archived tumor tissue. Every sample showed at least one affected gene, accounting for actionable mutations in 45 of 52 patients (87%). New treatment options that would not have been used as indicated by standard predictive markers (such as hormonal receptor status or HER2-status) were found in 22 of 52 patients (42%). We detected therapeutic relevant pathogenic germline variants in 9,6% (5/52) of the patients.

Conclusions: Using a high throughput-panel sequencing approach to identify actionable mutations in patients with metastatic breast cancer, we identified potential target-related treatment options in a large proportion of our patients, some of which would not have been considered without this data. Prospective clinical trials with compounds targeting the identified actionable mutations are needed to determine which treatments can indeed improve survival or quality of life by limiting exposure to ineffective drugs in advanced breast cancer.

## INTRODUCTION

Breast cancer is the most frequent cancer disease among woman. Although prognosis of patients with early breast cancer has improved over the last decades, metastatic breast cancer remains incurable [1]. Despite increasing therapeutic options regarding systemic treatment, predicting efficacy of a targeted drug on a patient level is still challenging. Although patients are faced with seemingly identical clinical and pathological diagnoses, their tumor genome, transcriptome, proteome, metabolome, the tumor environment, microbiome, patient’s immune system, and many other factors highly differ [2].

While sequencing technologies have made dramatic advances, the implementation of sequencing results into a routine clinical setting remains highly awaited. For this purpose, focused panel sequencing has major advantages as compared to research driven whole genome/exome/transcriptome analyses due to higher sensitivity and coverage. A gene panel test can be used to identify genetic alterations that are actionable by a distinct target-related drug but may also be used to identify mutations conferring drug resistance [3]. Moreover, mutational profiling can identify patients for “off label” use of approved compounds as well as for clinical trials where a distinct somatic mutation is part of the inclusion criteria [4–6]. Regarding breast cancer it is, however, not clear whether a large sequencing panel approach beyond the known biomarkers can actually aid decision-making.

Here, we performed a retrospective analysis of advanced breast cancer patients that underwent next-generation sequencing using a panel that covers more than 600 genes (latest version 742 genes). The size of the panel with greater than 2 megabases allows reliable calculation of tumor mutational burden. The aim of our study was to determine (i) the frequency of actionable mutations per patient and (ii) to analyze whether the respective new treatment suggestions are already approved for breast or other types of cancer or available within clinical trials.

## RESULTS

### Patient characteristics

A total of 52 patients were included in this study. Of those, 37 (71%) were ER/PR-positive and HER2-negative, 10 (19%) were HER2-positive and 6 (12%) were triple-negative at primary diagnosis (Table 1). Half of the patient tumors (*n* = 26, 50%) were classified as high grade (G3). Eleven (21%) of the patients had a primary metastatic disease. Tumor tissue for sequencing studies was collected from the primary tumor in 40 (77%) patients and from metastatic lesions in 12 (23%) patients.

**Table 1:** Patient characteristics (n = 52) based on primary tumor

		***n***	**%**
ER	Positive	42	80%
	Negative	10	19%
PR	Positive	40	77%
	Negative	12	23%
HER-2	Positive	10	19%
	Negative	42	81%
Grading	1	1	2%
	2	25	48%
	3	26	50%
M1 at initial diagnosis	Yes	11	21%
	No	41	78%

### Mutational spectrum

**Figure 1:**
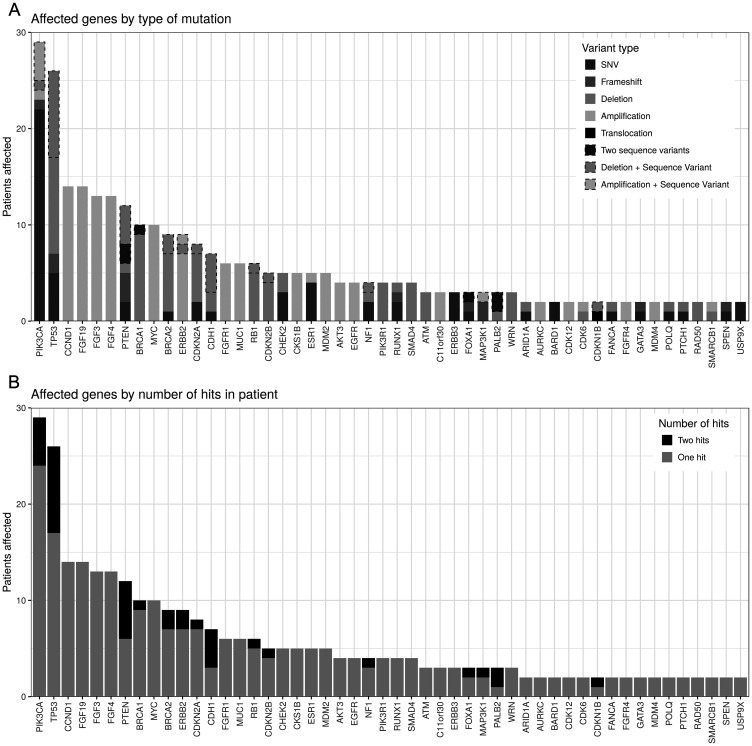
Genes with reported mutations order by number of patients affected. (**A**) By number of hits. In most patients, genes are only affected by a single mutation, while some genes, notably *TP53, PTEN* and *CDH1* are often affected by two hits (e.g., SNV and deletion). (**B**) By type of mutation. Dashed borders indicate double-hit cases (see top panel). *PIK3CA* is mostly affected by missense SNVs while *TP53* has a large number of deletions reported.

First we looked at the mutational spectrum and compared our data to what has been published so far [7]. We detected therapeutic relevant pathogenic/likely pathogenic germline variants in 9,6% (5/52) of the patients. Genes harbouring pathogenic/likely pathogenic mutations were namely *BRCA1, PALB2, TP53, MLH1* and *MSH3*. The most frequent somatic mutations affected the *PIK3CA* gene, followed by mutations in *TP53* and *CCND1* (Figure 1). The NGS analysis allows us to also detect copy number variation (CNV), increasing the number of detectable mutations. For example 26 patients had relevant alterations in *TP53*, of which 17 patients had a heterozygous deletion of *TP53*, 9 of them with a sequence alteration in the remaining allele. A further 9 patients had only sequence variants and no CNV affecting *TP53*. Without CNV analysis, 31% of patients with relevant *TP53* mutations would have been diagnosed as *TP53* wild type.

Next, we looked at tumor mutational burden (TMB). The TMB (somatic coding mutations per Megabase) was on average 2.9 Mut/Mb in our cohort (range 0–24). Only three patients had TMB > 5 Mut/Mb with values at 10, 12 and 24 Mut/Mb.

### Therapeutic options

Every tumor sample investigated showed at least one affected gene and the highest number of genes affected in one patient was 10. The following potential therapeutic options were identified (Table 2): AKT inhibitor (2% of all patients), PI3K inhibitor (35% of all patients), HER2-targeted therapy (23% of all patients), PARP inhibitor (15% of all patients), CDK4/6 inhibitor (15% of all patients), mTOR inhibitor (15% of all patients) and FGFR inhibitor (2% of all patients). In total, actionable mutations were found in 45 of 52 patients (87%). Of these, 73% harbored mutations where target-related therapy suggestions were already approved. For the remaining patients the identified treatments were available in clinical trials.

**Table 2:** Availability of the target-related drugs

Targeted-related therapy	Patients (*n*)	Patients (%)	availability
AKT inhibitor	1	1,9%	Phase III
PI3K inhibitor	18	34,6%	approved
Her2 targeted	12	23,1%	approved
PARP inhibitor	15	28,8%	approved
CDK4/6 inhibitor	7	13,5%	approved
mTOR inhibitor	8	15,4%	approved
FGFR inhibitor	1	1,9%	Phase II

Patients with multiple target-related therapeutic options appear in each of the respective rows.

To evaluate whether the results of genetic testing added value to clinical decision-making, we were interested which of the identified target-related drugs would not have been identified by using routine predictive biomarker (hormonal-receptor status and HER2-status). 10 of the 12 patients where amplification of HER2 was found using panel sequencing were HER2-positive (immunohistochemistry and/or fluorescence *in situ* hybridization of the primary tumor) and of the 15 cases where mTOR or CDK4/6 inhibition was identified as a potential treatment option 13 were hormonal-receptor positive and HER2-negative. In total, new treatment options were therefore found in 22 of 52 patients (42%).

## DISCUSSION

To guide treatment decision in precision oncology, panel sequencing techniques are powerful tools to identify actionable mutations in an individual tumor. We were interested whether the use of a gene panel that covers more than 600 genes potentially adds to the clinical information. The pathways that were mainly affected were the PI3K/AKT/mTOR pathway downstream of the growth factor receptor families e. g. FGFR/ERBB/EGFR. In total, 87% of our retrospective cohort harbored actionable alterations.

As reported previously, *PIK3CA* was the most frequently affected gene (35% of all patients [8]. Recently, findings from the SOLAR-I trial showed that the PI3Kα inhibitor alpelisib nearly doubles median progression free survival in hormonal receptor positive/HER2 negative breast cancer when given together with the antiestrogen fulvestrant to patients with *PIK3CA*-mutant disease while no effect was seen in patients without a *PIK3CA* mutation [9].

Furthermore, we frequently found an amplification of the locus containing *CCND1, FGF3, FGF4*, and *FGF19* on chromosome 11. Although it is hypothesized from *in vitro* studies that *CCND1* expression might predict response to CDK4/6 inhibitor treatment, biomarker analyses from the PALOMA-2 and MONALEESA-2 trial did not reveal any differences with respect to efficacy of palbociclib or ribociclib in patients with low versus high expression of CCND1 [10, 11]. Due to low toxicity but high and long-lasting response rates of CDK4/6 inhibition, these drugs should be used in all patients with hormonal receptor positive and HER2 negative advanced breast cancer until more reliable biomarkers become available [12–15].

We frequently observed *PTEN* and *BRCA1* loss in our cohort. This is of note as the types of mutations in these two genes are mainly deletions or a double hit (combination of a deletion with a deleterious variant) and might therefore have been overlooked in previously published data due to technical limitations in the detection of copy number variants. Additionally, *BRCA2* was frequently deleted. Recently, the EMBRACA and the OlympiaD trials have demonstrated that PARP inhibitor treatment using olaparib or talazoparib improves progression-free survival as compared to standard chemotherapy in advanced breast cancer patients that harbor a germline *BRCA1* or *BRCA2* mutation [16, 17].

Although EMBRACA and OlympiaD demonstrated efficacy of PARP inhibitor treatment only for germline *BRCA1/2* mutated patients, accumulating evidence suggests that other forms of dysfunctional homologous recombination repair, including somatic mutations in *BRCA1/2, ATM, ATR, PALB2,* or *CHEK2* are potential biomarkers for PARP inhibitor efficacy [18, 19].

The IMPACT/COMPACT study recently found at least one somatic mutation in 48% of patients with metastatic breast cancer. The authors compared survival of those patients that could be matched on genotype-matched trials versus those treated on non-genotype-matched trials. They found no differences with respect to median time on treatment, however, the results were biased by the availability of a respective trial and by the limited number of patients that were enrolled in a clinical trial and therefore available for final analysis [20]. In the prospective randomized SHIVA trial, 741 patients with advanced solid cancer of any type were randomly assigned to receive standard treatment according to physicians choice versus an experimental regime based on molecular profiling [21]. The authors found no difference with respect to clinical outcome. In the MOSCATO-01 trial an actionable mutation was found in 49% of the patients. Of note, only 19% of the patients suffered from metastatic breast cancer. Interestingly, of the 23% of all patients that were treated with targeted therapy matched to a genomic alteration, about one third had a PFS that was prolonged as compared to PFS on prior therapy [22].

Several issues are challenging with respect to the identification of tumor based genetic markers. First, in our cohort as well as in many translational research projects, archival tissue was used which in many cases originates from the primary tumor. This is crucial as tumor biology changes at disease progression and the genotype as well as the phenotype of metastatic tissue may differ from the primary tumor [23, 24]. Additionally, different metastatic sites may even differ when they are sampled at the same timepoint. The use of circulating DNA might alleviate this problem as it displays not only one metastatic site and can be easily reassessed during the course of therapy. Due to the small sample size we were not able to analyze our data within different subgroups of distinct therapy lines. Importantly, it was recently shown in the SOLAR-I trial that the *PIK3CA* mutation status as determined by the use of circulating tumor DNA predicts response to alpelisib [25]. Second, single DNA mutations are only a small part of the whole picture. Future investigations should therefore not only focus on the interplay of various mutations but also take gene expression on RNA or protein level, epigenetic changes as well as immunological and clinical factors into consideration. The combination of different therapies will be essential to overcome resistance and to address tumor heterogeneity. Huge amounts of clinical and molecular data will, however, be essential to address these questions [2].

This study has several limitations. First, the patient cohort is very small and our analysis had a retrospective design. Second, we have variability in the clinical situation, time point of tissue sampling, and time point of sequencing. Third, no data on the efficacy of targeted-related treatment and no follow-up data are available.

## MATERIALS AND METHODS

### Patients

Patients who underwent treatment for advanced or metastatic breast cancer at the Department of Gynecology and Obstetrics (University of Tuebingen, Germany) with available panel sequencing results were eligible for this retrospective analysis. The analysis was approved by the ethics committee of the University of Tuebingen (reference number: 234/2017B02).

### Sequencing of normal and tumor tissue

Sequencing of archived formalin-fixed paraffin-embedded tumor tissue (latest biopsy from a metastatic lesion or, if metastatic tissue was not available, primary tumor tissue) and blood as normal control was performed after written informed consent according to the gene diagnostics law in Germany. All panel sequencing results and the respective target related therapy suggestions were performed by CeGaT and Praxis fuer Humangenetik (Tuebingen, Germany). In brief, genomic DNA was isolated according to the manufacturers’ instructions using QIAamp DNA Blood Maxi Kit on QiaSymphony (Qiagen, Hilden, Germany). DNA quantity and quality were determined using Qubit^®^uFluorometer and NanoDrop ND-8000 (Thermo Fisher Scientific, Dreieich, Germany). For all patients, > 600 genes were analyzed (Supplementary Table 1). All coding regions and flanking intronic regions were enriched using Agilent in-solution technology with custom-design target regions. For sequencing, we used either the Illumina HiSeq2500, HiSeq4000, or NovaSeq6000 systems.

### Data processing and evaluation

Sequencing reads were demultiplexed using Illumina bcl2fastq (1.8.2). Adapter sequences were removed with Skewer 0.1.116 and the trimmed reads mapped to the human reference genome (hg19) using the Burrows Wheeler Aligner (BWA-mem 0.7.2). Reads mapping to more than one location with identical mapping scores were discarded. Duplicates resulting from PCR amplification were removed (samtools 0.1.18). Variants were called using samtools and varscan (2.3.3). Technical artifacts were removed (in-house software, CeGaT Tuebingen) and the remaining variants were annotated based on several internal and external databases. Mutations were defined as “actionable” if the variant causes a well known or high probability of functional protein change which secondly influences signaling pathways in a way in which its consequence can be therapeutically targeted. The knowledge of functional impact of the detected mutations as well as drugability is based on internal and external databases as well as literature search at the timepoint of data analysis. Copy number variations were computed using an internally developed method based on sequencing coverage depth (CeGaT Tuebingen, [26]). For each patient, both tumor tissue as well as healthy tissue were analyzed and the data compared to reliably distinguish somatic mutations from germline variants. To compute tumor mutational burden (TMB), first the somatic variants affecting the protein-coding regions (CDS) of all sequenced genes (both synonymous as well as non-synonymous) with a minimum variant frequency (VAF) of 10% were counted. Variants were split into driver and passenger mutations and the resulting two counts used to estimate the number of somatic variants in the whole exome. For this estimation, passenger mutations were assumed to occur with equal density in all known genes, i.e., their number was scaled up relative to the difference between gene panel size and whole exome size. Driver mutations were assumed to be limited to tumor-associated genes, and their number was not scaled up. The estimated total count of both passenger and driver mutations was normalized to the size of the complete coding exome.

Target related therapy suggestions were discussed within an interdisciplinary team of clinicians and molecular biologists (molecular tumor board).

## CONCLUSIONS

Large panel gene sequencing is feasible and affordable in a clinical setting and in our cohort 45 of 52 patients (87%) could be identified with targetable mutations. Of those, 22 patients could be presented with a novel therapeutic option not identified by routine biomarker analysis (42% of our cohort). Next to the identification of a large number of target related genes large panel gene sequencing may help to prioritize therapy lines of approved compounds, and to identify patients suitable for clinical trials. To better understand the relationship between driver mutation and treatment efficacy, molecular, clinical, and follow-up data should be recorded systematically. For this purpose, it is important that large gene panel sequencing is performed in accredited laboratories and results are discussed within interdisciplinary molecular tumor boards where the genetic information is interpreted together with the patient’s individual clinical context. Finally, it is of utmost importance to conduct large randomized clinical trials that address the question how individual patients can derive the most benefit from these novel technologies.

## SUPPLEMENTARY MATERIALS


