# Supplementary material for: Sequencing for an interdisciplinary molecular tumor board in patients with advanced breast cancer: experiences from a case series
Source: Oncotarget. 2020 Sep 1;11(35):3279–85. doi: 10.18632/oncotarget.27704 (PMC7476733; doi:10.18632/oncotarget.27704)
Supplement: Supplementary file 1 [file oncotarget-11-3279-s001.pdf]

Sequencing for an interdisciplinary molecular tumor board in patients with advanced breast cancer: experiences from a case series

SUPPLEMENTARY MATERIALS

Supplementary Table 1: Genes analyzed in this cohort

ABCB1*, ABCC2*, ABCG2*, ABL1, ABL2, ABRAXAS1, ACD*, ACVR1*, ACVR1B*, ACVR2A*, ADAMTS20*, ADGRA2, ADGRB3*, ADGRL3*, AFF1*, AFF3*, AIP*, AJUBA*, AKAP9*, AKT1, AKT2, AKT3, ALK, AMER1*, ANKRD26*, APC, AR, ARAF, ARFRP1*, ARHGAP26*, ARHGAP35*, ARID1A, ARID1B, ARID2, ARID5B*, ARNT*, ASXL1, ASXL2*, ATF1*, ATG2B*, ATM, ATP1A1*, ATP5F1B*, ATR, ATRX, AURKA, AURKB, AURKC, AXIN1, AXIN2, AXL, AZGP1*, B2M*, BABAM2*, BAP1, BARD1, BCL10, BCL11A, BCL11B, BCL2, BCL2L1*, BCL2L2*, BCL3, BCL6, BCL9, BCOR, BCORL1, BCR, BIRC2, BIRC3, BIRC5, BLM, BLNK*, BMPR1A, BRAF, BRCA1, BRCA2, BRD3*, BRD4*, BRIP1, BTK, BTNL2*, BUB1B, C11ORF30, CALR*, CAMK2G*, CARD11, CASP8*, CBFB, CBL, CBLB*, CBLC*, CCDC6, CCND1, CCND2, CCND3, CCNE1, CD1D*, CD274*, CD38*, CD52*, CD58*, CD70*, CD79A, CD79B, CD82, CDC27*, CDC73, CDH1, CDH11*, CDH2, CDH20*, CDH5*, CDK12, CDK4, CDK6, CDK8, CDKN1A*, CDKN1B, CDKN1C*, CDKN2A, CDKN2B, CDKN2C, CDX2*, CEBPA, CEP57*, CHD1*, CHD2*, CHD4*, CHEK1, CHEK2, CIC, CIITA*, CKS1B, CMPK1*, CNOT3*, COL1A1, COMMD1*, CRBN*, CREB1, CREBBP, CRKL, CRTC1, CRTC2*, CSF1R, CSF2*, CSF3R*, CSMD1*, CSMD3*, CSNK1A1*, CTCF, CTLA4*, CTNNA1, CTNNB1, CUL3*, CUL4B*, CUX1*, CXCR4*, CYLD, CYP1A1*, CYP1A2*, CYP2A6*, CYP2A7*, CYP2B6*, CYP2C19*, CYP2C8*, CYP2C9*, CYP2D6*, CYP2E1*, CYP3A4*, CYP3A5*, DAXX, DCC, DDB2, DDIT3*, DDR1*, DDR2, DDX11*, DDX3X*, DDX41*, DEK, DHFR*, DIAPH1*, DICER1, DIS3*, DIS3L2*, DKC1*, DNM2*, DNMT1*, DNMT3A, DOT1L, DPYD, DST*, EBP*, ECT2L*, EGFR, EGLN1*, EGR2*, EGR3*, ELAC2, ELANE*, ELF3*, EML4, ENG*, EP300, EP400*, EPAS1*, EPCAM, EPHA2*, EPHA3, EPHA4*, EPHA5*, EPHA7*, EPHB1*, EPHB4, EPHB6, EPHX1*, ERBB2, ERBB3, ERBB4, ERCC1, ERCC2, ERCC3, ERCC4, ERCC5, ERG, ERRFI1*, ESR1, ESR2*, ETNK1*, ETS1, ETV1, ETV4, ETV5, ETV6, EWSR1, EXO1*, EXT1, EXT2, EZH1*, EZH2, FAM123B*, FAN1*, FANCA, FANCB, FANCC, FANCD2, FANCE, FANCF, FANCG, FANCI, FANCL, FANCM, FAS, FAT1, FBXO11*, FBXW7, FES, FGF10, FGF14, FGF19, FGF2*, FGF23, FGF3, FGF4, FGF5*, FGF6, FGFBP1*, FGFR1, FGFR2, FGFR3, FGFR4, FH, FKBP1A*, FLCN, FLI1, FLT1, FLT3*, FLT4, FN1*, FOXA1*, FOXA2*, FOXE1*, FOXL2, FOXO1, FOXO3, FOXP1, FOXP4*, FOXQ1*, FRK*, FRS2*, FUBP1, FUS*, FYN*, FZR1*, G6PD, GABRA6*, GALNT12*, GATA1, GATA2, GATA3, GATA4*, GATA6*, GDNF*, GID4*, GLDN*, GLI1*, GLI2*, GNA11, GNA13, GNAQ, GNAS, GOPC*, GOT1*, GPC3, GPER1*, GREM1*, GRIN2A, GRM3*, GRM8*, GSK3A*, GSK3B*, GSTM1*, GSTP1*, GSTT1*, GUCY1A2*, GUSB*, H3F3A, H3F3B*, HCAR1*, HCK*, HGF, HIF1A, HIP1*, HIST1H3B, HLA-A*, HLA-B*, HLA-C*, HLA-DPA1*, HLA-DPB1*, HLA-DQA1*, HLA-DQB1*, HLA-DRA*, HLA-DRB1*, HLF, HMGA2*, HMGN1*, HMOX2*, HNF1A, HNF1B, HOOK3*, HOXA9*, HOXB13, HOXD8*, HRAS, HSD3B1*, HSP90AA1, HSP90AB1, ICK*, ID3*, IDH1, IDH2, IFNGR1*, IFNGR2*, IGF1R, IGF2, IGF2R, IKBKB, IKBKE, IKZF1, IKZF3*, IL1B*, IL1RN*, IL2, IL21R, IL6*, IL6ST, IL7R, ING1*, ING4, INHBA*, INPP4B*, INPPL1*, IRF1*, IRF2*, IRF4*, IRF6*, IRS2, ITGA10*, ITGA9*, ITGB2*, ITGB3*, ITK, JAK1, JAK2, JAK3, JUN, KAT6A, KAT6B*, KCNJ5*, KDM5A, KDM5C, KDM6A, KDR, KEAP1, KEL*, KIAA1549, KIT, KLF2*, KLF4*, KLF6*, KLHDC8B*, KLHL6, KMT2A*, KMT2B*, KMT2C*, KMT2D*, KNL1*, KRAS, LAMP1*, LATS1, LATS2, LCK, LGI1*, LIFR*, LIG4, LIMK2*, LMO1, LOC110117498-PIK3R3*, LPP*, LRP1B, LRRK2*, LTF*, LTK, LYL1*, LYN*, LZTR1*, MAD2L2*, MAF*, MAFB, MAGEA1, MAGI1*, MAGI2*, MALT1*, MAML1, MAML2*, MAP2K1, MAP2K2, MAP2K3*, MAP2K4, MAP2K5*, MAP2K6*, MAP2K7*, MAP3K1, MAP3K14*, MAP3K3*, MAP3K4*, MAP3K6*, MAP3K7*, MAPK1, MAPK11*, MAPK12*, MAPK3*, MAPK8*, MAPK8IP1*, MARK1*, MARK4*, MAX, MBD1, MC1R, MCL1, MDC1*, MDM2, MDM4, MECOM*, MED12, MEF2B, MEN1, MET, MGA*, MGMT*, MITF, MLH1, MLH3, MLL*, MLL2*, MLL3*, MLLT10, MLLT3, MMP2*, MN1, MOB1A*, MOB1B*, MPL, MPO*, MRE11, MS4A1*, MSH2, MSH3, MSH4*, MSH5*, MSH6, MSR1, MST1R*, MTHFR, MTOR, MTR*, MTRR, MUC1, MUC16*, MUTYH, MXI1, MYB, MYC, MYCL*, MYCL1*, MYCN, MYD88, MYH11, MYH9, NAT1*, NAT2*, NBN, NCOA1, NCOA2*, NCOA3*, NCOA4*, NCOR1*, NF1, NF2, NFE2L2, NFKB1, NFKB2, NFKBIA, NFKBIE*, NIN, NKX2-1*, NKX3-1*, NLRC5*, NLRP1*, NOP10*, NOTCH1, NOTCH2, NOTCH3, NOTCH4*, NPM1, NQO1*, NR1I3*, NRAS, NRG2*, NSD1, NSD2, NT5C2*, NTHL1*, NTRK1, NTRK2, NTRK3, NUMA1, NUP214*, NUP93*, NUP98, PAK3, PALB2, PALLD, PARP1*, PARP2*, PARP4*,

PAX3, PAX5, PAX7, PAX8*, PBK*, PBRM1, PBX1, PCBP1*, PDCD1*, PDCD1LG2*, PDE4DIP*, PDF*, PDGFA*, PDGFB, PDGFC*, PDGFD*, PDGFRA, PDGFRB, PDK1*, PER1*, PGAP3*, PGR*, PHF6, PHOX2B, PIAS4*, PIGA*, PIK3C2A*, PIK3C2B, PIK3C2G*, PIK3CA, PIK3CB, PIK3CD, PIK3CG, PIK3R1, PIK3R2, PIM1, PKHD1, PLAG1*, PLCG1, PLCG2*, PLEKHG5*, PML, PMS1, PMS2, POLD1, POLE, POLH, POLQ*, POLR3A*, POT1, POU2AF1*, POU2F2*, POU5F1*, PPARG*, PPM1D*, PPP2R1A*, PRDM1, PRDM16, PREX2*, PRF1, PRKACA*, PRKAR1A, PRKCA*, PRKCI*, PRKD1*, PRKDC, PRKN*, PROM2*, PRSS1, PRSS8*, PRX*, PSIP1, PSMB1*, PSMB10*, PSMB2*, PSMB5*, PSMB8*, PSMB9*, PSMC3IP*, PSPH*, PTCH1, PTCH2*, PTEN, PTGS2, PTK2*, PTK7*, PTPN11, PTPRC, PTPRD, PTPRT, QKI*, RAC1*, RAC2*, RAD21*, RAD50, RAD51*, RAD51B, RAD51C, RAD51D, RAD54B*, RAD54L*, RAF1, RALGDS, RARA, RARB*, RARG*, RASA1*, RASAL1*, RB1, RBM10*, RBM15*, RECQL*, RECQL4, REL, RET, RFC2*, RFX5*, RHBDF2*, RHEB*, RHOA*, RHOH*, RICTOR, RINT1*, RIPK1*, RIT1*, RNASEL, RNF2, RNF213*, RNF43, ROS1, RPL22*, RPL5*, RPS20*, RPS6KA2*, RPS6KB1*, RPTOR, RRM1*, RSF1*, RUNX1, RUNX1T1*, RXRA*, RYR1, SACS*, SAMD9*, SAMHD1*, SAV1, SBDS, SCG5*, SDHA, SDHAF2, SDHB, SDHC, SDHD, SEC23B*, SELP*, SEM1*, SEMA4A*, SEPT9*, SETBP1*, SETD2, SETDB1*, SF3B1, SGK1, SH2B1*, SH2B3*, SH2D1A, SH3GL1*, SHH*, SIK2*, SIN3A*, SIRT1*, SKP2*, SLC15A2*, SLC1A3*, SLC22A1*, SLC22A2*, SLC22A6*, SLC26A3*, SLCO1B1*, SLCO1B3*, SLIT2*, SLX4, SMAD2*, SMAD3, SMAD4, SMARCA4, SMARCB1, SMARCE1, SMC1A*, SMC3*, SMO, SMUG1*, SNCAIP*, SOCS1, SOS1*, SOX10*, SOX11, SOX17*, SOX2, SOX9*, SPEN*, SPINK1, SPOP, SPRED1*, SPTA1*, SRC, SRD5A2*, SRGAP1*, SRP72*, SRSF2, SSTR1*, SSTR2*, SSTR3*, SSTR5*, SSX1, STAG1*, STAG2, STAT1*, STAT3, STAT4*, STAT5A*, STAT5B*, STK11, STK11IP*, STK3*, STK36*, STK4*, SUFU, SULT1A1*, SUZ12*, SYK, SYNE1*, TAF1, TAF15, TAF1L*, TAL1*, TAP1*, TAP2*, TBK1*, TBL1XR1*, TBX22*, TBX3*, TCF12*, TCF3, TCF7L1*, TCF7L2, TCL1A, TEK*, TENT5C, TERC*, TERF2IP*, TERT*, TET1, TET2, TFE3, TGFBR2, TGM7*, THBS1*, TIMP3*, TLR4, TLX1, TLX3*, TMEM127, TMPRSS2*, TNF*, TNFAIP3, TNFRSF11A*, TNFRSF13B*, TNFRSF14, TNFRSF1A*, TNFRSF1B*, TNFRSF25*, TNFRSF8*, TNFSF11*, TNK2, TOP1, TOP2A*, TP53, TP53BP1*, TPMT*, TPR*, TPX2*, TRAF2*, TRAF3*, TRAF5*, TRAF6*, TRAF7*, TRIM24*, TRIM33*, TRIP11*, TRRAP, TSC1, TSC2, TSHR, TUBA4A*, TUBB*, TYMS, U2AF1, UBE2T*, UBR5, UGT1A1*, UGT2B15*, UGT2B17*, UGT2B7*, UIMC1, UNG*, USP34*, USP9X, VEGFA*, VEGFB*, VHL, VKORC1, WAS*, WASF3*, WISP3, WRN, WT1, WWTR1*, XIAP*, XPA, XPC, XPO1, XRCC1, XRCC2, XRCC3*, XRCC5*, XRCC6*, YAP1, ZBTB2*, ZFHX3, ZHX3*, ZNF217, ZNF384*, ZNF521*, ZNF703*, ZNRF3*, ZRSR2*

Three panel versions were used, containing 551 genes (8 patients), 649 genes (23 patients), and 711 genes (21 patients), respectively. 410 genes were analyzed in all patients, genes not analyzed in all patients are marked with a star. Gene names have been updated to reflect current HGNC nomenclature.
